# RNA-binding protein ELAVL1 modulates WDR36 to inhibit p53 pathway and reduce calcium overload in retinal cells under acute pressure elevation

**DOI:** 10.1515/biol-2025-1279

**Published:** 2026-04-20

**Authors:** QingFeng Meng, BaoLi Ning, WenXiong Chen, JingJing Zhang, Jun Fu

**Affiliations:** Health Center of Screening and Prevention of Diseases, First Affiliated Hospital of Harbin Medical University, Harbin City, Heilongjiang Province, 150001, China

**Keywords:** ELAVL1, WDR36, calcium overload, acute retinal injury, post-transcriptional regulation

## Abstract

Retinal ganglion cell (RGC) loss involves p53 activation and calcium overload. The role of RNA-binding proteins like ELAVL1 in this process remains unclear. We investigated whether ELAVL1 protects retinal cells by post-transcriptionally regulating WD Repeat Domain 36 (WDR36). *In vitro* stress was modeled using oxygen-glucose deprivation/reperfusion (OGD/R) in R28 retinal precursor cells. *In vivo* acute intraocular pressure (IOP) elevation was induced in mice. We modulated gene expression with siRNA, plasmids, or AAV vectors, assessing cell death, calcium levels, and protein markers. OGD/R downregulated ELAVL1 and WDR36 protein, but not WDR36 mRNA. ELAVL1 bound WDR36 mRNA and promoted its protein translation. Overexpression of either ELAVL1 or WDR36 reduced OGD/R-induced cell death, calcium overload, and p53 pathway activation. Crucially, WDR36 knockdown abolished ELAVL1’s protective effects *in vitro*. *In vivo*, AAV-mediated ELAVL1 overexpression mitigated IOP-induced retinal damage and apoptosis, which was reversed by co-knockdown of WDR36. The ELAVL1-WDR36 axis is a critical post-transcriptional mechanism that promotes retinal cell survival under acute pressure-ischemia stress by inhibiting p53 activation and calcium overload, highlighting its therapeutic potential.

## 1 Introduction

Degenerative changes and apoptosis of retinal ganglion cells (RGCs), which are key neurons for information transmission between the retina and the brain, are a major cause of blindness in a variety of ocular diseases such as glaucoma [1]. Glaucoma is the second leading cause of irreversible blindness worldwide and is characterized by progressive loss of RGCs and optic nerve atrophy, leading to visual field defects or even complete blindness [2]. Although elevated intraocular pressure (IOP) is considered a major risk factor for glaucoma, damage to RGCs may continue even when IOP is successfully controlled, suggesting that there are other key pathologic mechanisms besides IOP [3].

At the molecular level, injury and death of RGCs involve multiple complex cellular mechanisms, including oxidative stress, calcium overload, mitochondrial dysfunction, and activation of various cell death pathways [4], [5], [6]. In RGCs, the activation of the p53 pathway is acknowledged as a significant contributor to stress-related apoptosis, especially under ischemia-reperfusion injury and elevated intraocular pressure scenarios [7]. p53 can be rapidly activated when cells are stressed, which in turn regulates downstream target genes, ultimately leading to apoptosis [4], 8]. In addition, endoplasmic reticulum stress (ERS) and calcium homeostasis imbalance are also important mechanisms of RGC injury, and calcium overload triggers the activation of calcium-dependent proteases, destroys the cytoskeleton, and further exacerbates mitochondrial dysfunction in a deleterious cascade reaction [4], 9].

The role of post-transcriptional regulation in neuronal survival and functional maintenance has been increasingly emphasized. RNA-binding proteins (RBPs), as the core performers of post-transcriptional regulation, regulate RNA maturation, stability, subcellular localization, and translation efficiency by binding to specific RNA sequences [10], 11]. In the nervous system, the function of RBPs is particularly important because neurons are highly dependent on precise gene expression regulation to maintain their specialized functions [10], 12], 13]. However, although the importance of RBPs in the nervous system has been extensively studied, little is known about their specific roles in the stress response and survival of RGCs, especially in the setting of ischemic injury and elevated IOP.

ELAVL1 is a widely expressed RBP that regulates mRNA stability and translation mainly by binding to AU-enriched elements in the 3′ untranslated region (3′UTR) of target mRNAs [14]. In the nervous system, ELAVL1 is involved in the regulation of neuronal differentiation, synaptic plasticity and response to stress [13], 15]. ELAVL1 plays a protective role in cerebral ischemia-reperfusion injury, promoting neuronal survival by stabilizing specific mRNAs [16]. However, the function of ELAVL1 under retinal stress conditions and its molecular mechanisms have not been fully elucidated.

WD Repeat Domain 36 (WDR36) is a protein containing multiple WD40 repeat sequences that was originally identified as a susceptibility gene for primary open-angle glaucoma [17]. WDR36 is involved in fundamental cellular processes, and its dysfunction may lead to cellular stress and apoptosis [18], 19]. However, the specific role of WDR36 in the protection of retinal cells remains unclear, especially under stress conditions. Also, whether WDR36 regulates the survival of retinal cells by affecting the p53 pathway and calcium homeostasis has not been explored.

Notably, although p53 pathway activation and calcium overload are recognized as key mechanisms of death in RGCs, an in-depth understanding of the post-transcriptional regulatory dimensions of these pathological processes is lacking. In particular, it remains an unanswered question whether RBPs such as ELAVL1 affect the p53 pathway and calcium homeostasis by regulating the expression of specific target mRNAs (e.g., WDR36) at the post-transcriptional level, thereby influencing the survival of retinal cells under stress conditions. In addition, despite the recognized importance of post-transcriptional regulation in neuronal stress response, there is a relative paucity of relevant studies targeting RGCs, which limits the comprehensive understanding of the molecular mechanisms underlying retinal neurodegenerative diseases.

The present study aimed to investigate whether RBP ELAVL1 could protect retinal cells from acute stress injury by regulating WDR36, with a special focus on p53 pathway activation and calcium homeostasis imbalance, which are two key pathological mechanisms. By elucidating this novel post-transcriptional regulatory pathway, this study not only contributes to an in-depth understanding of the molecular mechanisms of retinal cell injury under acute IOP elevation, but also provides potential intervention targets for the treatment of acute IOP-induced retinal injury.

## 2 Materials and methods

### 2.1 Cell culture and oxygen glucose deprivation/reoxygenation (OGD/R) model

The R28 cell line (CP-R181, Pricella, Wuhan, China) is a retinal precursor cell line derived from postnatal day 6 rat retina immortalized with the 12S E1A gene. R28 cells exhibit certain RGC-like characteristics but are not primary retinal ganglion cells. The cells had been characterized for STR typing and regularly tested for mycoplasma contamination. R28 retinal precursor cells were cultured in high-glucose Dulbecco’s modified Eagle’s medium (DMEM, 11965092, Gibco, NY, USA) containing 10 % fetal bovine serum (FBS, 10099141, Gibco) and 1 % penicillin-streptomycin solution (15140122, Gibco). Cells were maintained in a standard cell culture incubator (37 °C, 95 % relative humidity with 5 % CO_2_). To establish the *in vitro* OGD/R model, logarithmic growth phase R28 retinal precursor cells were inoculated in 6-well plates (3516, Corning Inc., NY, USA) at 5 × 10^5^ cells/well and incubated for 24 h. Subsequently, the cells were washed twice with phosphate buffered saline (PBS, 10010023, Gibco) and put in glucose-free DMEM (11966025, Gibco). Cells were incubated in a triple gas incubator (HERAcell VIOS 160i, Thermo Fisher Scientific, MA, USA) for 4 h at 1 % O_2_, 5 % CO_2_, and 94 % N_2_. At the end of the hypoxic treatment, the sugar-free medium was replaced with normal medium and the cells were reoxygenated in a normoxic incubator (∼21 % O_2_, 5 % CO_2_) for 6 h. Control cells were continuously cultured in complete medium under normoxic conditions.

### 2.2 Cell transfection

Small interfering RNA (siRNA) targeting ELAVL1 and WDR36 (si-ELAVL1, si-WDR36) and negative control siRNA (si-NC) were purchased from RiboBio (Guangzhou, China). pcDNA3.1, pcDNA3.1-ELAVL1, pcDNA3.1-WDR36 overexpression vectors were synthesized by GENEWIZ (Nanjing, China). The siRNA (50 nM) or plasmid (2.5 µg) was transiently transfected into R28 retinal precursor cells grown to 70–80 % confluence using Lipofectamine 3000 transfection reagent (L3000015, Invitrogen, CA, USA). The efficiency of gene knockdown or overexpression was assessed by RT-qPCR and Western blot 48 h later. All *in vitro* experiments were performed with a minimum of three independent biological replicates.

### 2.3 CCK-8 test

R28 retinal precursor cells were inoculated in 96 wells at 2 × 10^3^ cells/well and adhered to the wall overnight. Then, 100 µL of fresh complete medium containing 10 µL of CCK-8 reagent (CK04, Dojindo, Japan) was added to each well. Incubation of the cells was continued for 2 h. Optical density values were measured at 450 nm using a multifunctional microplate reader (Molecular Devices SpectraMax M5, CA, USA). Each experiment included six technical replicates and was independently repeated three times.

### 2.4 Cytotoxicity assay

Cytotoxicity in R28 retinal precursor cells was determined using the LDH Cytotoxicity Assay Kit (Beyotime, Shanghai, China, Cat# C0016). The cell culture supernatant was mixed with LDH reaction working solution for 30 min at room temperature away from light. The absorbance at 490 nm was read using a microplate reader. Each experiment was independently repeated three times with triplicate wells.

### 2.5 Reactive oxygen species (ROS) assay

Intracellular ROS levels were measured using the fluorescent probe DCFH-DA (D6883, Sigma-Aldrich, MO, USA). R28 retinal precursor cells were inoculated at 6 × 10^5^ cells/well in 6-well plates, digested with trypsin, and resuspended in DMEM. DCFH-DA was added to a final concentration of 20 µM and incubated for 30 min at 37 °C protected from light. Analysis was performed using a flow cytometer (Beckman Coulter, CA, USA). Mean fluorescence intensity was analyzed using CytExpert software (Beckman Coulter). Each experiment was independently repeated three times.

### 2.6 Intracellular ATP assay

Total ATP levels in R28 retinal precursor cells were measured using the Bioluminescent Assay Kit (S0027, Beyotime, Shanghai, China). Cells were lysed by adding the lysis solution provided in the kit. ATP assay working solution was mixed with cell lysates, and luminescence values were measured using a multifunctional marker. Each experiment was independently repeated three times with triplicate wells.

### 2.7 Mitochondrial membrane potential (MMP) assessment

Changes in MMP were detected using the JC-1 Mitochondrial Membrane Potential Assay Kit (C2006, Beyotime). JC-1 is a cationic dye that accumulates in mitochondria in a membrane potential-dependent manner. In healthy cells with high MMP, JC-1 forms aggregates that emit red fluorescence (excitation/emission: 585/590 nm). In cells with depolarized mitochondria (low MMP), JC-1 remains as monomers that emit green fluorescence (excitation/emission: 514/529 nm). Cell suspensions prepared were incubated with JC-1 Staining Working Solution for 20 min at 37 °C and washed twice with JC-1 staining buffer (1×). Immediately analyzed using a flow cytometer (Beckman Coulter CytoFLEX S), changes in MMP were assessed by calculating the ratio of red fluorescence intensity to green fluorescence intensity, with a decrease in the ratio indicating a decrease in MMP. Each experiment was independently repeated three times.

### 2.8 Oxidative stress assay

Superoxide dismutase (SOD; A001-3-2), glutathione peroxidase (GSH-Px, A005-1-2), and malondialdehyde (MDA; A003-1-2) were detected in the cell lysates of R28 retinal precursor cells using commercial kits (Nanjing Jiancheng Bioengineering Institute, Nanjing, China) according to the manufacturer’s instructions. Each experiment was independently repeated three times.

### 2.9 Flow cytometry for calcium imaging

R28 retinal precursor cells were digested using 0.25 % Trypsin-EDTA solution (25200056, Gibco) and resuspended in Hanks’ Balanced Salt Solution (14025092, Gibco) containing Ca^2+^ and Mg^2+^ to set the cell density at 1 × 10^6^/mL. Fluo-4 AM Calcium Fluorescent Probe (F14201, Invitrogen) at 2 µM and Pluronic F-127 (P3000MP, Invitrogen) at 0.02 % were added, and the cells were incubated for 30 min at 37 °C away from light. Fluo-4 AM is a cell-permeant calcium indicator that exhibits increased fluorescence upon binding to intracellular Ca^2+^. Cells were immediately assayed on a flow cytometer (BD FACSCanto™ II, BD Biosciences, CA, USA) with excitation at 488 nm and emission collected at 530 nm. Mean fluorescence intensity, which is proportional to intracellular calcium concentration, was analyzed using FlowJo™ software (v10.8, BD Biosciences). Each experiment was independently repeated three times.

### 2.10 Western blot

Total protein was isolated from R28 retinal precursor cells using pre-cooled radioimmunoprecipitation assay cell lysis buffer at 4 °C and quantified using a bicinchoninic acid kit (20201ES76, Yeasen, Shanghai, China). Proteins were separated by polyacrylamide gel electrophoresis, transferred to polyvinylidene difluoride membranes (Millipore, MA, USA), and closed with 5 % bovine serum albumin for 1 h at room temperature. Subsequently, the membranes were incubated with primary antibodies at 4 °C overnight and re-probed with horseradish peroxidase-coupled secondary antibody (ab6721, Abcam) for 1 h at room temperature. The bands were visualized with an enhanced chemiluminescence system (Thermo Fisher Scientific) and analyzed with ImageJ software (version 1.50b). Primary antibodies used were: ELAVL1 (#12582, Cell Signaling Technology), p53 (#2527, Cell Signaling Technology), phospho-p53 (Ser15) (#9284, Cell Signaling Technology), p21 (#2947, Cell Signaling Technology), MDM2 (#86934, Cell Signaling Technology), Bax (#2772, Cell Signaling Technology), cleaved Caspase-3 (#9661, Cell Signaling Technology), CHOP (#2895, Cell Signaling Technology), GRP78/BiP (#3177, Cell Signaling Technology), ATF4 (#11815, Cell Signaling Technology), p-PERK (Thr980) (#3179, Cell Signaling Technology), p-IRE1α (Ser724) (ab124945, Abcam), GAPDH (#5174, Cell Signaling Technology), and WDR36 (#H00134430-M01, Novus Biologicals). Western blot experiments were independently repeated three times.

### 2.11 RNA immunoprecipitation (RIP)

RIP experiments were performed using the Magna RIP™ RNA-Binding Protein Immunoprecipitation Kit (17–700, Millipore). Briefly, approximately 1 × 10^7^ R28 retinal precursor cells were lysed with buffer and cell lysates were incubated overnight at 4 °C with magnetic beads conjugated to either anti-ELAVL1 antibody (12582, CST) or normal rabbit IgG (12–370, Millipore) as a negative control. After washing the magnetic bead complexes, the proteins were removed using proteinase K digestion. RNA was extracted and purified. Enrichment of WDR36 mRNA was detected by RT-qPCR, with GAPDH mRNA serving as a negative control target to verify the specificity of ELAVL1 binding. All RIP-qPCR results were normalized to Input samples. RIP experiments were independently repeated three times.

### 2.12 Protein stability analysis

R28 retinal precursor cells transfected with pcDNA3.1-ELAVL1 or pcDNA3.1 empty vector were treated with 100 μg/mL of Cycloheximide (C7698, Sigma-Aldrich) to inhibit *de novo* protein synthesis. Cells were harvested at 0, 2, 4, and 6 h after CHX addition and subjected to Western blot analysis to assess WDR36 protein half-life.

### 2.13 Acute IOP elevation-induced retinal injury mouse model

Male C57BL/6J mice (8 weeks of age, 18–22 g), totaling 40, were housed in SPF grade conditions (22 ± 2 °C, humidity 50 ± 10 %, 12 h light/dark) with free access to standard chow and water.

Referring to a previous study [20], an acute IOP elevation-induced retinal injury mouse model was constructed using anterior chamber perfusion. This model induces acute elevation of IOP to 90 mmHg for 60 min, simulating combined pressure-ischemia injury to the retina. It is important to note that this acute injury model is suitable for investigating the molecular mechanisms of acute IOP-induced retinal damage but does not fully recapitulate the pathological processes of chronic glaucoma, which involves progressive RGC degeneration over extended periods.

Briefly, mice were anesthetized by intraperitoneal injection with 100 mg/kg ketamine and 10 mg/kg xylazine mixture. Pupils were dilated using 1 % tropicamide and 2.5 % phenylephrine eye drops. Corneal surface anesthesia was performed with 0.5 % proparacaine hydrochloride eye drops. The anesthetized mice were placed on a thermostatic heating pad (37 °C), the left anterior chamber of the eye was carefully punctured with a 30G needle attached to saline (0.9 % NaCl) reservoir. The intraocular pressure (monitored with a TonoLab rebound tonometer, iCare Finland Oy, Vantaa, Finland) was raised to 90 mmHg and maintained for 60 min. Care was taken to keep the cornea moist during this period. The needle was withdrawn after 60 min to allow the IOP to return to normal. Control (Sham) mice underwent corneal puncture only without IOP elevation.

**Ethical approval:** The research related to animal use has been complied with all the relevant national regulations and institutional policies for the care and use of animals, and has been approved by the Institutional Animal Care and Use Committee of First Affiliated Hospital of Harbin Medical University (No. KY2024-114).

### 2.14 Gene intervention

To intervene with ELAVL1 and WDR36 expression *in vivo*, mice received intravitreal injections 3 weeks before the establishment of the acute IOP elevation model. This timing was chosen based on the following rationale: AAV2 serotype typically requires 2–4 weeks to achieve stable and sufficient transgene expression in retinal tissues following intravitreal delivery; pre-treatment ensures that ELAVL1 expression levels have reached experimental requirements before injury occurs; and this preventive intervention strategy is consistent with standard experimental paradigms in mechanistic studies within the field.

Recombinant adeno-associated virus (rAAV) vectors (serotype AAV2, VectorBuilder Inc., Guangzhou, China) carrying shRNA sequences targeting ELAVL1 or WDR36 (sh-ELAVL1, sh-WDR36) or negative control shRNA sequences (sh-NC) or overexpression sequences carrying ELAVL1 or WDR36 (OE-ELAVL1, OE-WDR36) or empty vector control (Vector) were used. AAV2 serotype has been reported to predominantly transduce the retinal ganglion cell layer following intravitreal injection, although transduction of other retinal cell types may also occur.

Using a micro syringe pump (World Precision Instruments, Sarasota, FL, USA) and a Hamilton micro syringe with a 33G needle, 1 µL of viral suspension (1 × 10^13^ GC/mL) was slowly injected into the vitreous of the left eye approximately 1 mm behind the corneoscleral limbus. Antibiotic eye ointment was applied after injection to prevent infection. Each experimental group included 8 mice (*n* = 8 per group).

### 2.15 Hematoxylin-Eosin (HE) staining

Seven days after acute IOP elevation treatment, mice were euthanized by CO_2_ inhalation overdose, and the eyes were immediately removed. The eyes were fixed in 4 % PFA solution at 4 °C for 48 h, subjected to gradient ethanol dehydration, xylene clearance, and paraffin embedding. Serial sections of 4 µm thickness were made using a rotary slicer (Leica RM2235, Wetzlar, Germany), dewaxed, and stained with HE (G1120, Solarbio, Beijing, China). The stained sections were dehydrated, cleared, sealed with neutral gum, and imaged using an orthopantominal light microscope (Nikon Eclipse Ni–U, Tokyo, Japan).

### 2.16 TUNEL staining

TUNEL staining was performed using the *In Situ* Apoptosis Detection Kit (11684795910, Roche Diagnostics, Mannheim, Germany). Briefly, 4 µm thick paraffin sections were deparaffinized, treated with proteinase K (P2308, Sigma-Aldrich, 20 μg/mL in PBS) for 15 min at 37 °C, and treated with 0.1 % Triton X-100 in 0.1 % sodium citrate solution on ice for 2 min. TUNEL reaction mixture was incubated protected from light for 1 h at 37 °C, followed by DAPI staining for 10 min. The sections were sealed with an antifluorescence quencher and observed under a fluorescence microscope (Nikon Eclipse Ni–U). TUNEL-positive cells in the ganglion cell layer were counted.

### 2.17 Data analysis

All data were expressed as mean ± standard deviation (SD). *In vitro* experiments were performed with at least three independent biological replicates (*n* = 3), and *in vivo* experiments included 8 mice per group (*n* = 8). Statistical analysis was performed using GraphPad Prism 9.0 (GraphPad Software, CA, USA). Two-group comparisons were performed using the Wilcoxon Rank-Sum Test. Multiple-group comparisons were performed using the Kruskal–Wallis H Test, followed by Dunn’s Test. Statistical differences were defined as *P*-value < 0.05.

## 3 Results

### 3.1 WDR36 overexpression inhibits p53 activation and calcium overload in R28 retinal precursor cells

**Figure 1:**
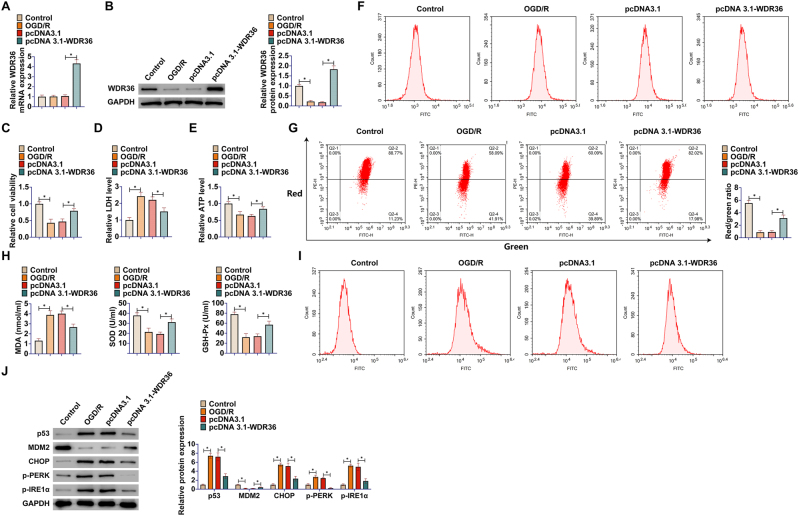
Overexpression of WDR36 significantly inhibits p53 activation and calcium overload in R28 retinal precursor cells. OGD/R model was established in R28 retinal precursor cells and pcDNA3.1-WDR36 was transfected into cells to upregulate the expression of WDR36. (A) RT-qPCR to detect WDR36 mRNA; (B) Western blot to detect WDR36 protein expression; (C) CCK-8 for cell viability of R28 retinal precursor cells; (D) Commercial kit for LDH release from R28 retinal precursor cells; (E) Commercial kit for ATP levels in R28 retinal precursor cells; (F) Flow cytometry for ROS levels in R28 retinal precursor cells using DCFH-DA probe; (G) Flow cytometry for MMP in R28 retinal precursor cells using JC-1 staining, with the ratio of red to green fluorescence reflecting changes in mitochondrial membrane potential; (H) Commercial kit for MDA, SOD and GSH-Px levels; (I) Flow cytometry to detect intracellular calcium ion levels in R28 retinal precursor cells using Fluo-4 AM probe, with mean fluorescence intensity proportional to intracellular Ca^2+^ concentration; (J) Western blot to detect protein expression of p53, MDM2, CHOP, p-PERK (Thr980), and p-IRE1α (Ser724) in R28 retinal precursor cells. Data are expressed as mean ± SD (*n* = 3 independent experiments). *P < 0.05.

To investigate the role of WDR36 in OGD/R-induced R28 retinal precursor cell injury, the effects of OGD/R treatment and exogenous WDR36 expression on endogenous WDR36 were first evaluated. RT-qPCR results showed that OGD/R treatment by itself did not significantly alter the mRNA levels of WDR36; however, transfection with pcDNA3.1-WDR36 successfully upregulated its mRNA expression (Figure 1A). Interestingly, OGD/R treatment significantly decreased the protein expression level of WDR36, while overexpression of WDR36 effectively restored its protein level (Figure 1B). On this basis, the effect of WDR36 overexpression on OGD/R-induced cellular damage was further evaluated. CCK-8 assay showed that OGD/R significantly reduced the viability of R28 retinal precursor cells, whereas overexpressing WDR36 significantly elevated the survival of damaged cells (Figure 1C). Consistent with this, LDH release assays showed that WDR36 overexpression significantly reduced OGD/R-induced cell membrane damage (Figure 1D) and partially restored intracellular ATP levels (Figure 1E).

Mitochondrial dysfunction and calcium homeostasis were further evaluated. The results showed that OGD/R treatment induced a dramatic increase in intracellular ROS levels (Figure 1F) and a significant decrease in MMP (Figure 1G), which was accompanied by an increase in MDA, an indicator of oxidative stress, as well as a decrease in SOD and GSH-Px (Figure 1H). Overexpression of WDR36 was able to significantly reverse these oxidative stress responses and mitochondrial dysfunction induced by OGD/R. It was observed that OGD/R treatment led to a significant increase in intracellular calcium ion levels, which was effectively inhibited by WDR36 overexpression (Figure 1I). Western blot analysis further revealed that OGD/R treatment significantly upregulated p53, CHOP, p-PERK, and p-IRE1α proteins, suggesting that the p53 pathway and the ERS pathway were activated, whereas overexpressing WDR36 significantly suppressed the upregulation of these key proteins (Figure 1J), and may mediate the regulation of p53 by affecting MDM2 (Figure 1J). These results suggest that overexpression of WDR36 in R28 retinal precursor cells effectively attenuated OGD/R-induced cellular injury, and its protective effects were closely related to the inhibition of oxidative stress, maintenance of mitochondrial function, attenuation of calcium overload, and suppression of the activation of p53 and ERS signaling pathways.

### 3.2 ELAVL1 post-transcriptionally regulates WDR36 protein levels in R28 retinal precursor cells

**Figure 2:**
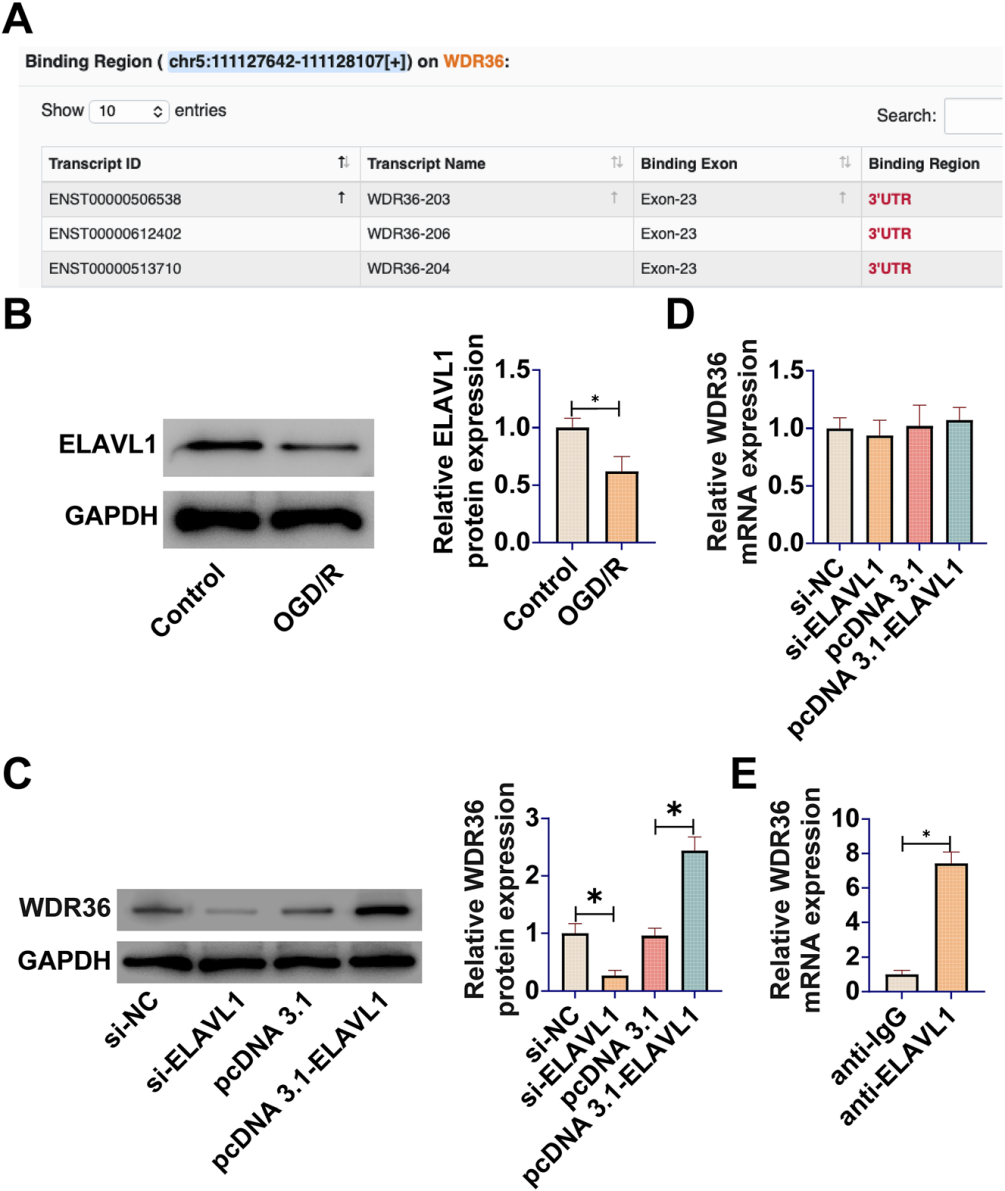
ELAVL1 post-transcriptionally regulates WDR36 protein levels in R28 retinal precursor cells. (A) Potential binding sites between ELAVL1 and WDR36 mRNA predicted using the bioinformatics platform StarBase; (B) Western blot to detect ELAVL1 protein expression in the OGD/R model; (C) Western blot to detect WDR36 protein expression in R28 retinal precursor cells following ELAVL1 knockdown or overexpression; (D) RT-qPCR to detect WDR36 mRNA level; (E) Interaction between ELAVL1 and WDR36 mRNA in R28 cells verified by RNA immunoprecipitation (RIP) assay (IgG as the negative antibody control, GAPDH mRNA as the negative target gene control, enrichment normalized to Input samples, *P* < 0.05); (F) RIP assay to confirm the interaction between ELAVL1 and WDR36 mRNA in R28 retinal precursor cells. IgG served as negative control antibody, and GAPDH mRNA served as negative control target to verify binding specificity. Enrichment was normalized to Input samples; (G) Western blot to detect WDR36 protein expression following ELAVL1 overexpression. Data are expressed as mean ± SD (*n* = 3 independent experiments). *P < 0.05.

Given the downregulation of WDR36 protein levels and relatively stable mRNA levels observed in the OGD/R model, it was hypothesized that its expression may be subject to post-transcriptional regulation. To explore this mechanism, RBPs that may interact with WDR36 mRNA were first predicted using the bioinformatics tool StarBase, and a potential binding site was found between ELAVL1 and WDR36 mRNA (Figure 2A). Interestingly, Western blot analysis showed that ELAVL1 protein expression also showed a significant reduction in OGD/R-treated R28 retinal precursor cells, which was consistent with the trend of WDR36 protein (Figure 2B).

To determine whether ELAVL1 directly regulates WDR36 expression, ELAVL1 levels in R28 retinal precursor cells were manipulated by knockdown and overexpression experiments. The results showed that ELAVL1 knockdown significantly reduced the protein abundance of WDR36, whereas ELAVL1 overexpression effectively elevated WDR36 protein levels (Figure 2C). Importantly, neither ELAVL1 knockdown nor overexpression significantly affected the mRNA level of WDR36 (Figure 2D), which strongly supports the hypothesis that ELAVL1 regulates WDR36 at the post-transcriptional level.

To further validate the direct interaction between the two, RIP experiments were performed. Compared with the IgG control group, the ELAVL1 antibody significantly enriched WDR36 mRNA, while the negative control gene GAPDH mRNA showed no significant enrichment (Figure 2F), suggesting that ELAVL1 can form a complex with WDR36 mRNA. It should be noted that RIP experiments demonstrate the physical association between ELAVL1 and WDR36 mRNA under *in vivo* conditions, but whether this binding is direct or indirect, as well as the specific binding sites, requires further verification using higher-resolution techniques such as CLIP-seq.

Finally, to investigate the mechanism by which ELAVL1 regulates WDR36 protein levels, the effect of ELAVL1 overexpression on WDR36 protein expression was assessed. Western blot analysis showed that ELAVL1 overexpression significantly increased WDR36 protein levels (Figure 2G). Taken together, these data suggest that ELAVL1 positively regulates WDR36 protein expression at the post-transcriptional level by binding to WDR36 mRNA.ELAVL1 affects p53 activation and calcium overload in R28 retinal precursor cells.

**Figure 3:**
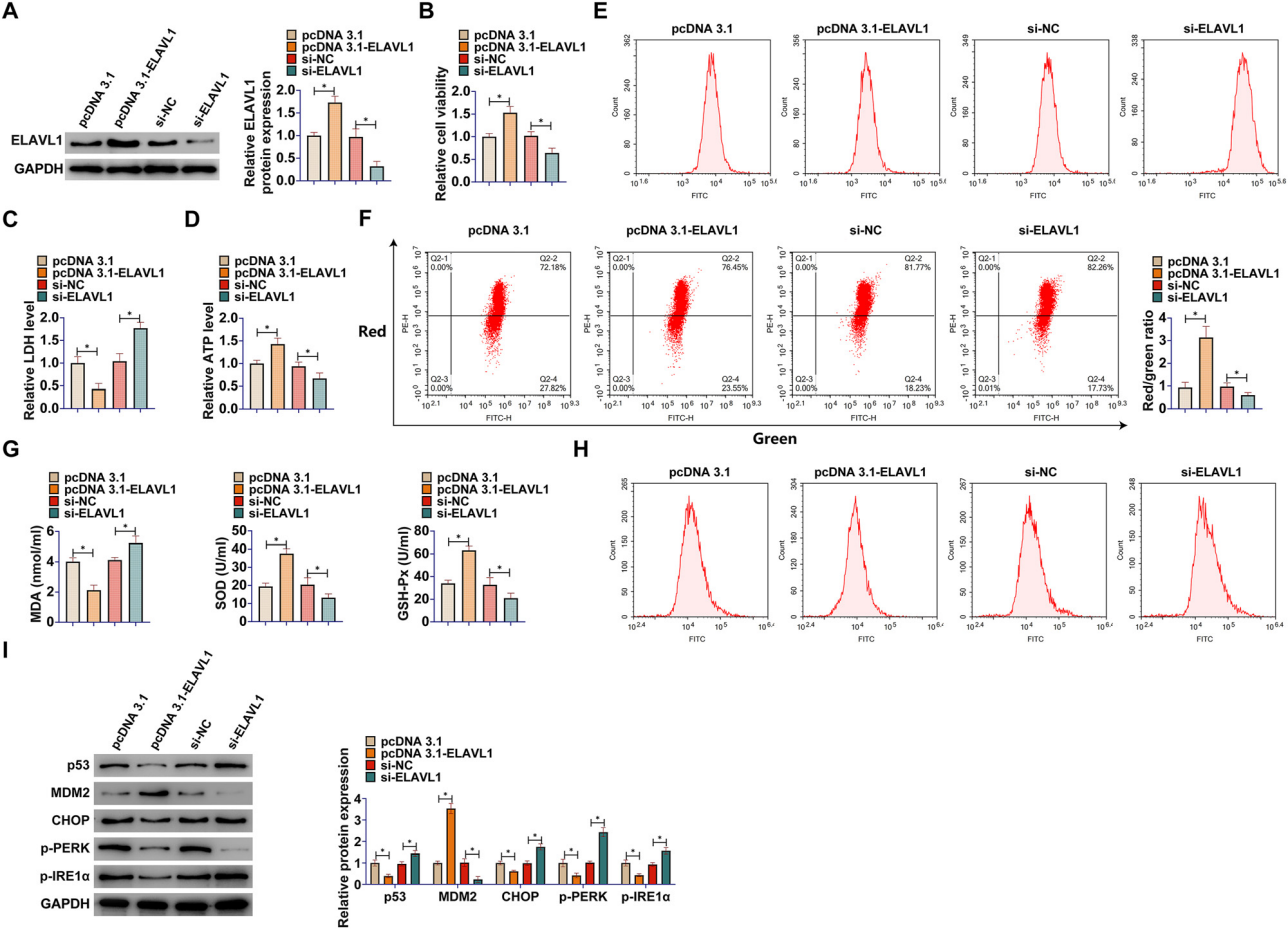
ELAVL1 affects p53 activation and calcium overload in R28 retinal precursor cells. The OGD/R model was established in R28 retinal precursor cells and pcDNA3.1-ELAVL1 or si-ELAVL1 was transfected into the cells to upregulate or knock down ELAVL1 expression. (A) Western blot to detect ELAVL1 protein expression after transfection; (B) CCK-8 for cell viability of R28 retinal precursor cells; (C) Commercial kit for LDH release from R28 retinal precursor cells; (D) Commercial kit for ATP levels in R28 retinal precursor cells; (E) Flow cytometry for ROS levels in R28 retinal precursor cells; (F) Flow cytometry for MMP in R28 retinal precursor cells using JC-1 staining; (G) Commercial kit for MDA, SOD and GSH-Px levels; (H) Flow cytometry to detect intracellular calcium ion levels in R28 retinal precursor cells using Fluo-4 AM probe; (I) Western blot to detect protein expression of p53, MDM2, CHOP, p-PERK (Thr980), and p-IRE1α (Ser724) in R28 retinal precursor cells. Data are expressed as mean ± SD (*n* = 3 independent experiments). *P < 0.05.

The functional role of ELAVL1 itself in the OGD/R-induced R28 retinal precursor cell injury model was next directly explored. ELAVL1 overexpression and knockdown were first successfully achieved in OGD/R-treated R28 retinal precursor cells by transfection with pcDNA3.1-ELAVL1 or si-ELAVL1 (Figure 3A). Functional experiments showed that ELAVL1 overexpression significantly enhanced the survival of R28 retinal precursor cells under OGD/R conditions (Figure 3B), reduced the release of LDH (Figure 3C), and increased the intracellular ATP level (Figure 3D), suggesting that ELAVL1 has a role in protecting the cells against OGD/R injury. Conversely, knockdown of endogenous ELAVL1 exacerbated the OGD/R-induced decreased cell viability (Figure 3B), increased LDH release (Figure 3C), and ATP depletion (Figure 3D).

Further mechanistic analyses indicated that ELAVL1 directly affected the oxidative stress status and mitochondrial function of cells. Specifically, ELAVL1 overexpression significantly inhibited OGD/R-induced ROS accumulation (Figure 3E), stabilized the MMP (Figure 3F), decreased MDA content, and elevated SOD and GSH-Px (Figure 3G); whereas the opposite effect, i.e., aggravation of oxidative stress and mitochondrial damage, was observed by knocking down ELAVL1 (Figure 3E–G). In addition, ELAVL1 overexpression was effective in attenuating the OGD/R-induced elevation of intracellular calcium ion concentration (Figure 3H), whereas ELAVL1 knockdown resulted in more severe calcium overload (Figure 3H). Finally, Western blot results showed that ELAVL1 overexpression significantly inhibited the OGD/R-induced upregulation of p53, CHOP, p-PERK, and p-IRE1α protein levels, whereas ELAVL1 knockdown further enhanced the expression of these proteins (Figure 3I), suggesting that ELAVL1 is involved in the regulation of p53 and the ERS signaling pathway. Altogether, these data clearly demonstrate that modulation of ELAVL1 expression in R28 retinal precursor cells can directly influence cellular injury, oxidative stress, mitochondrial function, calcium homeostasis, and the activation of related signaling pathways under OGD/R conditions.

### 3.3 ELAVL1 inhibits p53 activation and calcium overload in R28 retinal precursor cells by targeting WDR36

**Figure 4:**
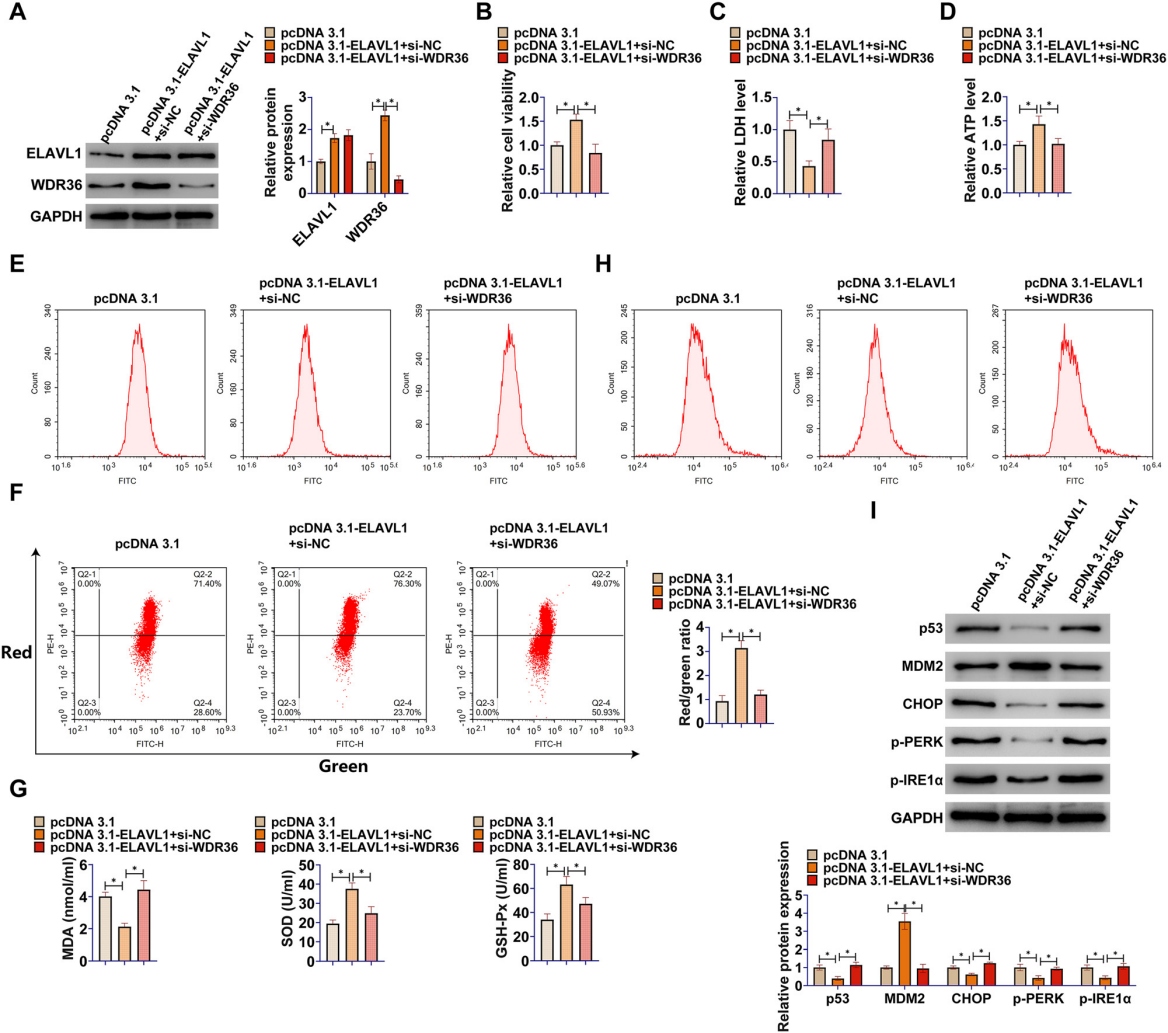
ELAVL1 inhibits p53 activation and calcium overload in R28 retinal precursor cells by targeting WDR36. The OGD/R model was established in R28 retinal precursor cells and pcDNA3.1-ELAVL1 and si-WDR36 were co-transfected into the cells. (A) Western blot to detect ELAVL1 and WDR36 protein expression after transfection; (B) CCK-8 for cell viability of R28 retinal precursor cells; (C) Commercial kit for LDH release from R28 retinal precursor cells; (D) Commercial kit for ATP levels in R28 retinal precursor cells; (E) Flow cytometry for ROS levels in R28 retinal precursor cells; (F) Flow cytometry for MMP in R28 retinal precursor cells; (G) Commercial kit for MDA, SOD and GSH-Px levels; (H) Flow cytometry to detect intracellular calcium ion levels in R28 retinal precursor cells; (I) Western blot to detect protein expression of p53, MDM2, CHOP, p-PERK (Thr980), and p-IRE1α (Ser724) in R28 retinal precursor cells. Data are expressed as mean ± SD (*n* = 3 independent experiments). *P < 0.05.

To further corroborate whether the protective function of ELAVL1 is dependent on its regulation of WDR36, rescue experiments were designed and executed. OGD/R-treated R28 retinal precursor cells were co-transfected with pcDNA3.1-ELAVL1 and si-WDR36. Western blot analysis showed that WDR36 expression was elevated in the pcDNA3.1-ELAVL1 + si-NC group, whereas in the pcDNA3.1-ELAVL1 + si-WDR36 group, WDR36 protein level was effectively knocked down (Figure 4A).

Functional analyses showed that concurrent knockdown of WDR36 significantly reversed the beneficial effects of ELAVL1 overexpression. Specifically, knockdown of WDR36 reversed the elevated cell viability (Figure 4B), increased LDH release (Figure 4C), and reduced ATP levels (Figure 4D) that were improved by ELAVL1 overexpression. Similarly, in terms of oxidative stress and mitochondrial function, knockdown of WDR36 significantly counteracted the ameliorative effects of ELAVL1 overexpression on ROS production (Figure 4E), MMP stabilization (Figure 4F), and MDA, SOD, and GSH-Px levels (Figure 4G). Especially critical, knockdown of WDR36 likewise reversed the inhibitory effect of ELAVL1 overexpression on intracellular calcium overload (Figure 4H). Western blot results further confirmed that the deletion of WDR36 significantly impaired the ability of ELAVL1 overexpression to inhibit the upregulation of p53, CHOP, p-PERK, and p-IRE1α proteins (Figure 4I). These results demonstrated that the protective effects of ELAVL1 on R28 retinal precursor cells under OGD/R conditions, including key aspects such as inhibition of p53 activation and calcium overload, were largely achieved by maintaining the expression and function of its downstream target gene WDR36.

### 3.4 ELAVL1 ameliorates retinal injury in the acute IOP elevation model by targeting WDR36

**Figure 5:**
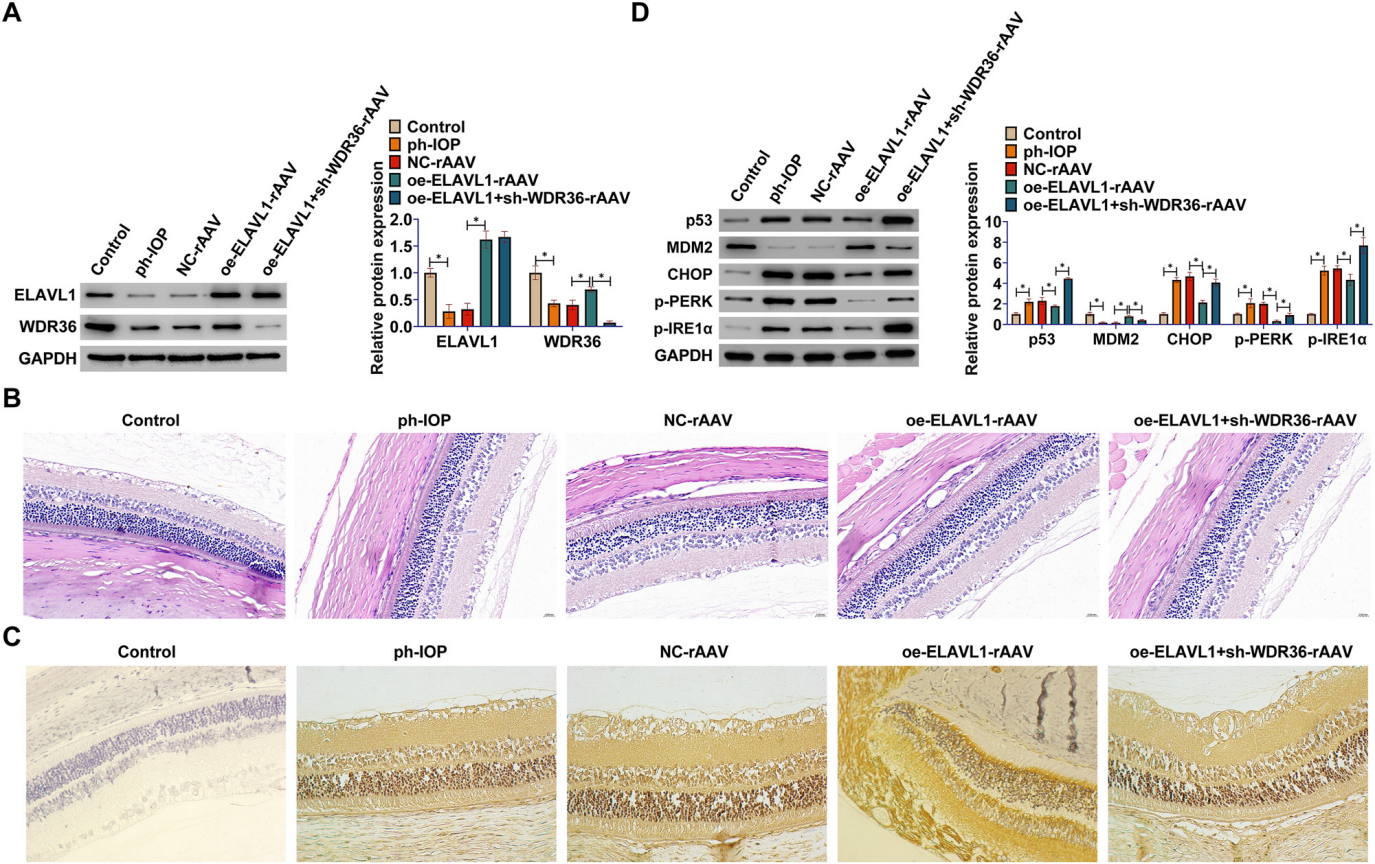
ELAVL1 ameliorates retinal damage in the acute IOP elevation model by targeting WDR36. An acute IOP elevation-induced retinal injury mouse model was established and gene intervention was performed using overexpression or knockdown rAAV vectors targeting ELAVL1 and WDR36. AAV vectors were delivered via intravitreal injection 3 weeks before model establishment. (A) Western blot to detect the protein expression of ELAVL1 and WDR36 in the retinal tissues of mice in each group; (B) HE staining to detect retinal tissue damage; (C) TUNEL staining to detect apoptotic cells in retinal tissues of mice in each group, with TUNEL-positive cells counted in the ganglion cell layer; (D) Western blot to detect protein expression of p53, MDM2, CHOP, p-PERK (Thr980), p-IRE1α (Ser724) in retinal tissues of mice in each group. Data are expressed as mean ± SD (*n* = 8 mice per group). *P < 0.05.

An acute IOP elevation-induced mouse retinal injury model was subsequently employed and targeted gene intervention was performed using the rAAV vector. Western blot analysis confirmed the intervention effect: compared with the model group injected with NC-rAAV, injection of oe-ELAVL1-rAAV significantly increased ELAVL1 protein expression in retinal tissues and correspondingly upregulated WDR36 expression; whereas in the group co-injected with oe-ELAVL1-rAAV and sh-WDR36-rAAV, WDR36 protein expression was effectively suppressed (Figure 5A).

Histological analysis showed that acute IOP elevation treatment resulted in significant retinal structural disorganization and damage, which was significantly attenuated by rAAV-mediated ELAVL1 overexpression (Figure 5B). Further, TUNEL staining showed that ELAVL1 overexpression significantly reduced the number of apoptotic cells in the retinas of acute IOP elevation model mice (Figure 5C), suggesting an *in vivo* antiapoptotic effect.

To confirm whether the *in vivo* protective effect of ELAVL1 was dependent on WDR36, the effect of knocking down WDR36 in the context of ELAVL1 overexpression was assessed. The results showed that knockdown of WDR36 (oe-ELAVL1 + sh-WDR36-rAAV group) significantly reversed the retinal protection (Figure 5B) and antiapoptotic effects (Figure 5C) associated with ELAVL1 overexpression alone. Consistent with this, exploration of molecular mechanisms revealed that acute IOP elevation induced elevated levels of p53, CHOP, p-PERK, and p-IRE1α proteins in retinal tissues, and ELAVL1 overexpression effectively inhibited the upregulation of these proteins; however, this inhibitory effect was significantly attenuated when WDR36 was knocked down (Figure 5D). These data suggest that ELAVL1 plays a protective role in the acute IOP elevation-induced retinal injury model by targeting WDR36, effectively ameliorating retinal injury and inhibiting apoptosis and stress signaling pathway activation.

## 4 Discussion

A new post-transcriptional regulatory mechanism is identified in this research, emphasizing the vital role of the ELAVL1-WDR36 axis in protecting retinal cells under acute pressure-ischemia stress. Experimental data suggest that ELAVL1 regulates WDR36 protein levels at the post-transcriptional level by binding to WDR36 mRNA, which in turn coordinates multiple intracellular signaling networks to maintain mitochondrial function, calcium homeostasis, and inhibit p53-dependent cell death. This finding not only extends the understanding of the function of ELAVL1 in retinal cell protection, but also provides a potential target for the treatment of acute IOP-induced retinal injury.

Post-transcriptional regulation plays a central role in adaptive and pathological stress responses [21]. It was observed in the study that WDR36 protein was significantly reduced under OGD/R conditions while its mRNA level remained relatively stable, an inconsistency suggesting the involvement of post-transcriptional regulatory mechanisms. ELAVL1 usually stabilizes target mRNAs or affects their protein output by binding to the 3′UTR AU-enriched element (ARE) of mRNAs [22]. Interestingly, RIP experiments confirmed that ELAVL1 was able to bind WDR36 mRNA, which may represent a previously unreported post-transcriptional regulatory relationship. Although our data indicate that ELAVL1 regulates WDR36 protein expression through a post-transcriptional mechanism, the exact molecular mechanism – whether by directly promoting mRNA translation, enhancing mRNA stability, or both – remains to be elucidated through polysome profiling, luciferase reporter assays, or protein synthesis rate measurements in future studies [23], [24], [25].

Notably, ELAVL1 itself was also downregulated under OGD/R conditions, which may be due to enhanced hypoxia-induced autophagy leading to ELAVL1 protein degradation, or through a HIF-1α-dependent transcriptional inhibition mechanism. This suggests that the ELAVL1-WDR36 axis may be a sensitive regulator of the early stress response to ischemic injury, and that changes in its expression may trigger the breakdown of downstream protective mechanisms.

WDR36 is mainly involved in ribosome biosynthesis and rRNA processing [26]. In this work, WDR36 could regulate the p53 signaling pathway, which is consistent with a previous report [18]. WDR36 overexpression significantly reduced the OGD/R- and acute IOP elevation-induced increase in p53 protein levels, while increasing MDM2 expression. Given that MDM2 is a major negative regulator of p53 [27], [28], [29], our data suggest that WDR36 may promote ubiquitination and proteasomal degradation of p53 by stabilizing or enhancing MDM2 function. However, the precise molecular mechanism by which WDR36 regulates MDM2 was not directly examined in this study.

Notably, the ELAVL1-WDR36 axis was closely linked to the ERS response. OGD/R- and acute IOP elevation-induced phosphorylation of PERK and IRE1α as well as upregulation of CHOP were significantly inhibited by ELAVL1 or WDR36 overexpression. Our data show that both the p53 pathway and ERS pathway are regulated by the ELAVL1-WDR36 axis, but whether there is a direct regulatory relationship between these two pathways was not verified in this study. This suggests that WDR36 may be a key regulator of endoplasmic reticulum homeostasis [30]. In addition, WDR36 overexpression was experimentally observed to significantly reduce OGD/R-induced intracellular calcium overload, indicating a role in calcium homeostasis regulation.

Mitochondrial dysfunction is a central pathological mechanism in ischemic retinopathy. The present study demonstrated that the ELAVL1-WDR36 axis was critical for maintaining mitochondrial function. This protective effect may be realized through multiple interrelated pathways, including reduced outer mitochondrial membrane permeability transition by inhibiting p53 activation and improved calcium homeostasis that attenuates calcium-mediated mitochondrial damage [31], [32], [33], [34].

The present study has several limitations that should be acknowledged. First, we used R28 retinal precursor cells for *in vitro* experiments. Although R28 cells exhibit certain RGC-like characteristics, they are not primary retinal ganglion cells, and their properties may differ from those of mature RGCs. Therefore, *in vitro* findings need to be validated in primary RGCs or combined with *in vivo* experiments. Second, the experiments validated this mechanism mainly in the *in vitro* OGD/R model and the acute IOP elevation model, which is suitable for investigating the molecular mechanisms of acute IOP-induced retinal damage but does not fully recapitulate the pathological process of chronic glaucoma. The role of the ELAVL1-WDR36 axis in chronic glaucoma progression needs to be verified using chronic high IOP models such as DBA/2J mice or microbead-induced models. In addition, whether long-term pathological mechanisms involved in chronic glaucoma, such as axonal transport defects and neurotrophic factor deprivation, are similarly regulated by ELAVL1 remains to be investigated.

Third, although binding of ELAVL1 to WDR36 mRNA has been demonstrated by RIP, this technique demonstrates the physical association between proteins and RNA under *in vivo* conditions, but whether this binding is direct or indirect, and the specific binding sites, require further verification using higher-resolution techniques such as CLIP-seq. Fourth, this study employed AAV2 serotype for retinal gene delivery. Although AAV2 has been reported to predominantly transduce the retinal ganglion cell layer following intravitreal injection, transduction of other retinal cell types may also occur. This study did not employ RGC-specific promoters or perform systematic evaluation of cell-type-specific transduction efficiency; therefore, it cannot be ruled out that ELAVL1 overexpression or knockdown may affect other retinal cell types, and the observed protective effects may partly derive from indirect effects of non-RGC cells. Future studies could employ AAV vectors driven by RGC-specific promoters such as Thy1.2 or Brn3b to further define the specific function of ELAVL1 in RGCs.

Fifth, this study employed retinal tissue-level functional and morphological assessments to evaluate the protective effects of ELAVL1 but did not perform quantitative analysis using RGC-specific markers such as Brn3a or RBPMS. Although the retinal ganglion cell layer is the primary target cell population for acute IOP-induced injury, and the visual function protection and apoptosis inhibition effects we observed are consistent with improved RGC survival, it cannot be completely ruled out that ELAVL1 affects other retinal cell types. Future studies should combine RGC-specific marker staining or employ RGC conditional gene manipulation models to more precisely define the direct role of ELAVL1 in RGC protection.

Future studies should focus on exploring the following directions: identify the precise binding site of ELAVL1 on WDR36 mRNA using techniques such as CLIP-seq and validate its functional significance by mutational analysis; identify the interacting protein network of WDR36 using immunoprecipitation and mass spectrometry; validate the protective role of the ELAVL1-WDR36 axis in primary retinal ganglion cells and animal models of chronic glaucoma. Of particular interest, because ELAVL1 is a widely expressed RBP that regulates numerous target genes, future therapeutic strategies may need to specifically enhance ELAVL1 regulation of WDR36 without affecting other targets.

In conclusion, this study reveals that the ELAVL1-WDR36 axis acts as a key post-transcriptional regulator of retinal cell protection under acute pressure-ischemia stress, which maintains cell survival by modulating p53 signaling, ERS, calcium homeostasis, and mitochondrial function. This finding provides potential molecular targets for the development of novel protective strategies against acute IOP-induced retinal injury. Targeted intervention against the ELAVL1-WDR36 axis may represent a promising therapeutic strategy for acute IOP-related retinal damage, although its applicability to chronic glaucoma requires further investigation.
